# Functional Neurological Disorders in Children: A Clinical Case Series of 196 Patients in a National Specialist Service

**DOI:** 10.1192/bjo.2025.10115

**Published:** 2025-06-20

**Authors:** Isabella Conti, Paul Harris, Amin Elmubarak, Joyita Sinha, Benjamin Baig

**Affiliations:** ^1^South London and Maudsley NHS, London, United Kingdom; ^2^Kings College, London, United Kingdom

## Abstract

**Aims:** Functional Neurological Disorders (FND) are common but poorly understood causes of disabling symptoms including limb weakness, seizures, fatigue and memory difficulties. Children as young as 5 years old may suffer from the condition and middle adolescence is the most common age of onset. Children with FND are likely to have more difficulties with education and mental health disorders and have experienced more adversity in childhood such as physical, emotional, and sexual abuse. Despite the severity and chronicity of this condition, the literature about FND in children is poor. Children with FND often fall between paediatric and CAMHS services and thus understanding the condition can be better done through cases referred to specialist services. Here we aim to describe the clinical characteristics of referrals made to the sole National and Specialist paediatric liaison service which assesses and treats children with FND.

**Methods:** Through the South London and Maudsley NHS Foundation Trust, Electronic Patient Journey System, an electronic case record search was completed for all patients who were given a diagnosis of ICD–10 Dissociative Disorders which was made before the patients were 18 years old. From 2015–2025, a total of 196 patients were identified. Individual case records were assessed for demographic and clinical information.

**Results:** In the group of 196 patients, patients ranged from 7–17 years old (mean 14.11 and mode 15). Sixty-eight per cent (69) of the patients were female. Forty-nine (25%) were coded as dissociative motor disorder, 54 (27.6%) dissociative convulsions, 6 (3%) dissociative amnesia, 71 (36.2%) had mixed or unspecified dissociative symptoms. Sixty-eight per cent of patients reported comorbid anxiety symptoms and 29% of patients reported comorbid depression. Fifty-eight per cent reported experiencing bullying at school.

**Conclusion:** Children with FND present with a high degree of disability, psychiatric comorbidity and social adversity. A limited evidence base exists as to best practice for paediatric FND yet treatment is essential for positive outcomes in both childhood and subsequent adulthood. Further research should include controlled trials in this age group and an increase in funding specialist services.

